# Endoscopic Ultrasound-Guided Pancreatic Cystic Fluid Biochemical and Genetic Analysis for the Differentiation Between Mucinous and Non-Mucinous Pancreatic Cystic Lesions

**DOI:** 10.3390/jcm14113825

**Published:** 2025-05-29

**Authors:** Angelo Bruni, Luigi Tuccillo, Giuseppe Dell’Anna, Francesco Vito Mandarino, Andrea Lisotti, Marcello Maida, Claudio Ricci, Lorenzo Fuccio, Leonardo Henry Eusebi, Giovanni Marasco, Giovanni Barbara

**Affiliations:** 1 IRCCS Azienda Ospedaliero-Universitaria di Bologna, Policlinico S. Orsola, 40138 Bologna, Italy; angelo.bruni4@unibo.it (A.B.); luigi.tuccillo@studio.unibo.it (L.T.); claudio.ricci6@unibo.it (C.R.); lorenzo.fuccio3@unibo.it (L.F.); leonardo.eusebi@unibo.it (L.H.E.); giovanni.barbara@unibo.it (G.B.); 2 Department of Medical and Surgical Sciences, University of Bologna, 40138 Bologna, Italy; 3 Gastroenterology and Gastrointestinal Endoscopy Unit, IRCCS San Raffaele Hospital, Via Olgettina 60, 20132 Milan, Italy; dellanna.giuseppe@hsr.it (G.D.); mandarino.francesco@hsr.it (F.V.M.); 4 Gastroenterology and Gastrointestinal Endoscopy Unit, IRCCS Policlinico San Donato, Piazza Edmondo Malan 2, 20097 San Donato Milanese, Italy; 5 Gastroenterology Unit, Hospital of Imola, University of Bologna, 40126 Bologna, Italy; a.lisotti@ausl.imola.bo.it; 6 Department of Medicine and Surgery, University of Enna ‘Kore’, 94100 Enna, Italy; marcello.maida@unikore.it; 7 Gastroenterology Unit, Umberto I Hospital, 94100 Enna, Italy

**Keywords:** pancreatic cystic, intracystic glucose, mucinous differentiation

## Abstract

Pancreatic cystic lesions (PCLs) are increasingly identified via computerized tomography (CT) and magnetic resonance (MR), with a prevalence of 2–45%. Distinguishing mucinous PCLs (M-PCLs), which include intraductal papillary mucinous neoplasms (IPMNs) and mucinous cystic neoplasms (MCNs) that can progress to pancreatic ductal adenocarcinoma, from non-mucinous PCLs (NM-PCLs) is essential. Carcinoembryonic antigen (CEA) remains widely used but often demonstrates limited sensitivity and specificity. In contrast, endoscopic ultrasound-guided measurement of intracystic glucose more accurately differentiates PCL subtypes, as tumor-related metabolic changes lower cyst fluid glucose in mucinous lesions. Numerous prospective and retrospective studies suggest a glucose cut-off between 30 and 50 mg/dL, yielding a sensitivity of 88–95% and specificity of 76–91%, frequently outperforming CEA. Additional benefits include immediate point-of-care assessment via standard glucometers and minimal interference from blood contamination. DNA-based biomarkers, including KRAS and GNAS mutations, enhance specificity (up to 99%) but exhibit moderate sensitivity (61–71%) and necessitate specialized, expensive platforms. Molecular analyses can be crucial in high-risk lesions, yet their uptake is constrained by technical challenges. In practice, combining glucose assessment with targeted molecular assays refines risk stratification and informs the choice between surgical resection or active surveillance. Future investigations should establish standardized glucose thresholds, improve the cost-effectiveness of genetic testing, and integrate advanced biomarkers into routine protocols. Ultimately, these strategies aim to optimize patient management, limit unnecessary interventions for benign lesions, and ensure timely therapy for lesions at risk of malignant transformation.

## 1. Introduction

Pancreatic cystic lesions (PCLs) are increasingly identified due to the widespread application of advanced imaging modalities such as computed tomography (CT) and magnetic resonance imaging (MRI), with an estimated prevalence ranging between 2 and 45% of the general population [1]. These lesions exhibit a wide spectrum ranging from benign lesions, such as serous cystadenomas with minimal malignant potential, to premalignant and malignant entities, including mucinous cystic neoplasms (MCNs) and intraductal papillary mucinous neoplasms (IPMNs) [2]. The accurate characterization of PCLs is clinically relevant due to the poor prognosis of pancreatic ductal adenocarcinoma (PDAC), which has a reported five-year mortality rate of 89% [3,4]. Discriminating between lesions devoid of malignant potential and those that are premalignant or malignant remains critical to avoid overtreatment of benign cysts and to identify patients who require active surveillance or surgical resection [5,6]. A major challenge in this context is distinguishing between mucinous PCLs (M-PCLs) and non-mucinous PCLs (NM-PCLs), as most premalignant PCLs are mucinous [1,7,8]. Although, certain NM-PCLs, including cystic neuroendocrine tumors, solid pseudopapillary neoplasms, cystic metastatic epithelial neoplasms, and cystic ductal adenocarcinomas, also carry a substantial risk of malignant transformation [1,9,10,11].

Benign pancreatic lesions, such as pseudocysts and serous cystadenomas (SCNs), constitute 16–39% of all pancreatic cysts, with SCNs being almost universally benign [12]. Their prevalence increases when lesions are discovered incidentally during imaging performed for unrelated conditions [13,14].

**Figure 1:**
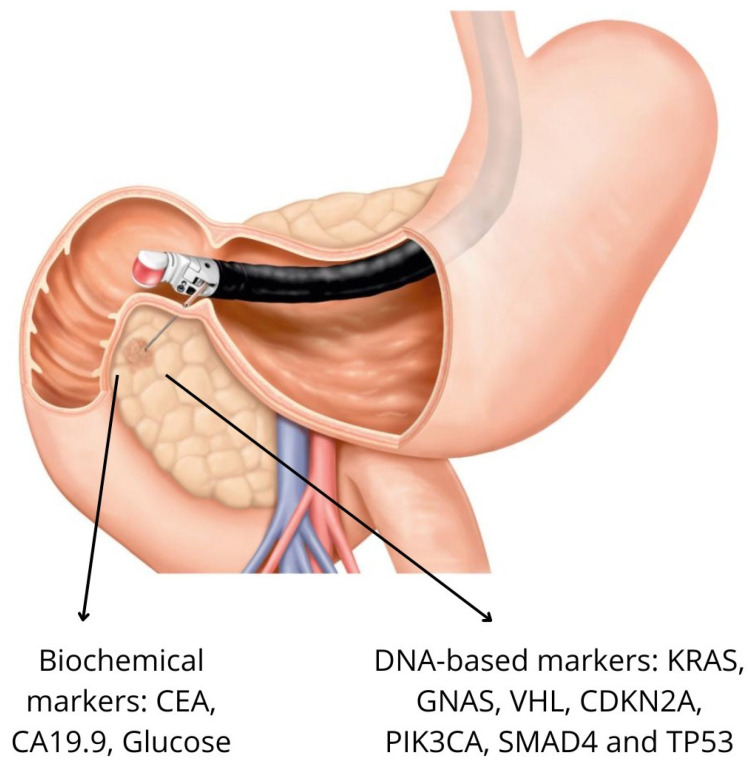
Illustration of endoscopic ultrasound-guided fine-needle aspiration (EUS-FNA) of a pancreatic cyst with biochemical markers (CEA, CA19-9, and glucose) and DNA-based markers (KRAS, GNAS, VHL, CDKN2A, PIK3CA, SMAD4, and TP53).

Among the premalignant PCLs, MCNs and IPMNs are the most frequent diagnoses. MCNs demonstrate a malignancy risk ranging from 1–34%, whereas IPMNs show variable malignant potential depending on subtype: branch-duct IPMNs carry a 12–47% risk, and main-duct IPMNs present a higher risk of 38–68% [14,15]. Although malignant progression remains infrequent in the broader population of PCLs, the 5–10-year risk is estimated at approximately 5–8%, with the risk increasing for main-duct IPMNs and lesions exhibiting worrisome features such as mural nodules or a cyst size greater than 3 cm [3]. Current guidelines recommend endoscopic ultrasound (EUS) evaluation for PCLs that exhibit worrisome features on cross-sectional imaging, with fine-needle aspiration (FNA) for cyst fluid analysis, cytological assessment, and biochemical marker evaluation (Figure 1) [2,16].

Cytological assessment primarily aims to identify mucinous epithelium, atypical cells, or overt malignancy, although sensitivity is often limited due to the rather low number of cells in the cyst fluid, reaching suboptimal accuracy in the context of FNA [17]. Biochemical cystic fluid measurements, including amylase, carcinoembryonic antigen (CEA), and glucose, play a central role in differentiating PCL subtypes. Elevated amylase suggests pancreatic duct communication—commonly seen in IPMN—while CEA is frequently used to distinguish mucinous pancreatic cystic lesions (M-PCLs) from non-mucinous pancreatic cystic lesions (NM-PCLs) [18].

The differential expression of biomarkers in PCLs derives from the underlying epithelial phenotype and metabolic activity. M-PCLs tend to harbor dysplastic or neoplastic mucin-producing epithelium with altered glycoprotein secretion and metabolic reprogramming, which influence the intralesional levels of tumor-associated antigens and glucose consumption [12].

Indeed, recently, intracystic glucose has emerged as a promising biomarker to support this differentiation. This review synthesizes the latest evidence on pancreatic intracystic biomarkers, with a specific focus on glucose, comparing its diagnostic performance to traditional markers, discussing its clinical applicability, and addressing the challenges associated with its integration into practice.

## 2. Diagnostic Utility of CA19.9

Serum carbohydrate antigen 19-9 (CA 19-9), also known as Sialyl Lewis-a, is a glycoprotein expressed on the surface of epithelial cells and requires the presence of the Lewis blood group antigen (specifically 1,4-fucosyltransferase) for its synthesis [19]. As a tumor-associated marker, CA 19-9 is the most widely used biomarker in pancreatic cancer, primarily for evaluating the risk of malignant transformation in PCLs. Its clinical utility stems from its overexpression in pancreatic ductal adenocarcinoma (PDAC) and other malignancies, where elevated serum levels may indicate neoplastic progression [20]. However, its diagnostic performance is limited by factors such as false-positive elevations in benign conditions (e.g., cholestasis, pancreatitis) and the inability to detect tumors in individuals who are genetically Lewis-negative (Lea-b-), as they cannot produce CA 19-9 [1,21,22]. International and European guidelines consider a serum CA 19-9 level >37 U/mL to be a “*worrisome feature*” and a “*relative indication for surgery*”, although not a standalone criterion for resection [1,23]. The American College of Gastroenterology also lists elevated CA 19-9 among high-risk features warranting EUS and FNA [18].

A meta-analysis evaluating serum CA 19-9 at a threshold of 37 U/mL found a pooled sensitivity of only 40%, a specificity of 89%, and an odds ratio of 4.34, indicating that a substantial proportion of pancreatic cancers will not be detected if this cut-off is applied [24]. Approximately 6–22% of individuals cannot produce CA 19-9, and additionally, some nonmalignant conditions (cholestasis, cirrhosis, hepatitis, diabetes, hypothyroidism) may also cause elevations [25]. Moreover, the marker appears more indicative of advanced disease than early neoplastic changes.

Recent prospective data from the PACYFIC registry show that the currently accepted CA 19-9 serum cut-off (>37 U/mL) does not accurately predict high-grade dysplasia (HGD) or invasive cancer (IC) [21]. Moreover, half of HGD/IC cases had normal values, and 60% of patients operated on for elevated CA 19-9 had benign disease. Although raising the cut-off to ≥133 U/mL improves specificity to 99%, it identifies only about 1% of progression cases, reflecting limited sensitivity. These findings are consistent with other studies: up to 72% of advanced cancers can have normal CA 19-9, and elevated values may indicate advanced rather than early disease [26,27].

While serum CA 19-9 provides systemic information, reflecting tumor burden or inflammation, its intracystic measurement was hypothesized to reflect local antigen secretion by cyst epithelium. However, this theoretical advantage has not been translated into diagnostic superiority.

Indeed, a recent meta-analysis assessing intracystic CA 19-9 concluded that it does not significantly improve diagnostic accuracy for differentiating M from NM cysts [27]. Despite initial expectations, sensitivity and specificity remain around 68%, inter-study heterogeneity is high, and there is no standardized cut-off. These findings indicate that intracystic CA 19-9 is no more reliable than CEA for diagnostic purposes and, overall, exhibits lower accuracy than CEA [26,27].

## 3. Diagnostic Utility of CEA

While the usefulness of serum CEA for pancreatic lesions is under controversy because of the high rate of false-positive results as a consequence of comorbid situations, such as diabetes, smoking habits, or colorectal polyps [28,29], intracystic CEA has traditionally served as a key biomarker for differentiating M from NM cysts, yet its diagnostic accuracy and optimal cut-off thresholds have been a subject of ongoing debate [30,31].

Initially identified as a fetal glycoprotein, CEA is minimally expressed in healthy adults; however, it can be upregulated in neoplastic settings [32]. Although the conventional intracystic 192 ng/mL threshold has been widely used, subsequent analyses, including a recent comprehensive 20-year institutional review, have revealed limitations in its applicability [33]. At this commonly applied cut-off, sensitivity may be as low as 51–56%, with specificity around 78%, diverging from initial expectations. Increasing the threshold to 250 ng/mL can improve specificity to the originally reported level of approximately 85%, although at the expense of a slight reduction in sensitivity [34,35].

A pooled analysis of 31 studies evaluating CEA revealed sensitivities of 67% and specificities of 80%, with an area under the receiver operating characteristic (ROC) curve of 0.79 [35,36].

These observations underscore the complexity of relying solely on CEA for clinical decision-making. Variability in reported optimal intracystic CEA thresholds—ranging from as low as 20 ng/mL to as high as 800 ng/mL—reflects differences in patient populations, study methodologies, and lesion histotypes [25]. Furthermore, elevated CEA is not entirely specific to mucinous lesions; certain non-mucinous cystic tumors, including some cystic neuroendocrine neoplasms, can also produce high CEA levels, occasionally leading to misclassification and unnecessary interventions [22,37,38]. Such heterogeneity necessitates a more accurate interpretation of CEA values, considering additional diagnostic tools and clinical-radiological context. As an example, molecular analyses (KRAS, GNAS, RNF43) and longer follow-up data have refined biomarker evaluation, yet no definitive threshold has emerged. A flexible approach, possibly adopting higher CEA cut-offs (e.g., 250 ng/mL) in specific scenarios to reduce false positives, remains necessary.

## 4. Diagnostic Utility of Intracystic Glucose Measurement

Glucose is an emerging biomarker in the diagnosis and understanding of the pathophysiology of PCLs. Tumor cells, including those in mucinous cysts, exhibit altered glucose metabolism via the Warburg effect, wherein glucose is preferentially metabolized to lactate even under aerobic conditions. This metabolic shift not only supports tumor growth and progression but also accounts for the observed glucose depletion in cyst fluid, which serves as a distinguishing feature of mucinous cysts [39].

In addition to the optimal diagnostic accuracy achieved through laboratory-based assessments, it has now been established that on-site evaluation using standard glucometers is both reliable and accurate for measuring glucose levels and risk stratification [40]. Glucose testing can be performed at the point-of-care location, providing immediate results that can influence clinical decisions without delay. Moreover, Zamir et al. have highlighted the cost-effectiveness of this approach, noting that on-site glucose testing is substantially less expensive than laboratory assays for CEA or other molecular markers [41].

Recent evidence shows that intracystic glucose dynamics differ markedly between the two mucinous subtypes. In IPMN, glucose levels cluster at very low values; thresholds below 30 mg/dL have produced area-under-the-curve (AUC) scores of up to 0.95, outperforming traditional biomarkers [42]. By contrast, MCNs display a wider dispersion; although many lesions remain hypoglycemic, intermediate values up to 80–90 mg/dL are not uncommon. In a prospective cohort dominated by MCNs, Zamir et al. achieved maximal diagnostic yield only after increasing the glucose cut-off to 87 mg/dL (sensitivity 91%, specificity 83%) [41].

Moreover, in a large multicenter analysis of 247 patients with M-PCLs, a glucose cut-off of <50 mg/dL achieved a sensitivity greater than 93% and a specificity of 77% [43]. Another evaluation of 108 patients identified an optimal threshold of 87 mg/dL, resulting in close to 91% sensitivity and 83% specificity, with an overall diagnostic accuracy nearing 90% [41]. Similar thresholds around 41 to 50 mg/dL have been reported in other populations, maintaining sensitivities and specificities in the range of 88% to over 90% [44].

Meta-analyses and several trials have confirmed that glucose cut-offs at or below 50 mg/dL provide excellent diagnostic reliability, with pooled sensitivity and specificity exceeding 88% [30,40,45,46].

This approach is particularly valuable in lesions yielding limited cyst fluid, such as small IPMNs or early-stage MCNs. Additionally, pooled analyses have consistently shown that a 50 mg/dL threshold optimally balances sensitivity and specificity. Table 1 presents a comparative overview of the diagnostic performance from multiple studies, detailing sensitivity, specificity, and the most effective cut-off values for accurately differentiating mucinous from non-mucinous pancreatic cystic lesions.

The comparative advantage of glucose is further supported by several individual studies that have refined its diagnostic thresholds [47,48]. For instance, in a French multicenter retrospective analysis, a glucose level below 41.8 mg/dL achieved sensitivity and specificity rates of 95.3% and 91.2%, respectively, outperforming the traditional CEA threshold of 192 ng/mL, which demonstrated lower sensitivity (41.7%) despite high specificity (96.9%) [49].

Additionally, two meta-analyses confirm glucose’s superiority over CEA, demonstrating higher sensitivity (91% vs. 56%; 90% vs. 63%) and comparable specificity (86–82% vs. 96–84%), resulting in superior diagnostic accuracy for distinguishing M-PCLs [50,51]. Notably, the meta-analyses confirmed that combining glucose with CEA did not enhance diagnostic performance, reinforcing the efficacy of glucose as a standalone biomarker.

**Table 1:** Diagnostic performance of different glucose and CEA cut-off values for distinguishing mucinous from non-mucinous PCLs.

Reference	Year	Study Design	No. of Patients	Type of Glucose Testing	Glucose Cut-Off for M-PCLs (mg/dL)	CEA Cut-Off for M-PCLs (mg/dL)	Glucose Sensitivity (%)	Glucose Specificity (%)	CEA Sensitivity (%)	CEA Specificity (%)
Zamir et al. [41]	2022	Prospective, single-center	101	Laboratory and glucometer	<87	>192	90.9	83.3	46.1	100.0
Zikos et al. [30]	2015	Retrospective, single-center	65	Laboratory and glucometer	<50	>192	95.0	57.0	77.0	83.0
Carr et al. [42]	2018	Prospective, single-center	153	Glucometer	<50	>192	92.0	87.0	58.0	96.0
Faias et al. [51]	2019	Retrospective, single-center	82	Glucometer	<50	>192	89.0	86.0	72.0	96.0
Ribaldone et al. [31]	2020	Prospective, single-center	56	Laboratory	<50	>192	93.6	96.0	54.8	100.0
Bruni et al. [40]	2024	Prospective, multicenter	50	Laboratory and glucometer	≤50	≥192	93.2	76.5	55.6	87.5
Simons-Linares et al. [34]	2020	Retrospective, single-center	113	Laboratory	≤41	≥192	92	92	51	88
Rossi et al. [45]	2020	Prospective, single-center	48	Laboratory	≤30	≥192	91.3	100.0	37.5	100.0
Yadav et al. [52]	2014	Retrospective, single-center	17	Laboratory	<21	>184	100.0	83.3	36.45	100.0

However, CEA retains value in scenarios requiring high specificity but with reduced sensitivity. Emerging evidence positions glucose, unaffected by diabetes in cited studies, as a more reliable first-line biomarker for mucinous cysts, often achieving better accuracy than CEA and complementing its diagnostic utility.

## 5. DNA-Based Biomarkers

DNA-based biomarkers for pancreatic cyst diagnosis are molecular markers derived from genetic material that can provide valuable and useful information about the nature of pancreatic cysts.

The recently introduced PancreaSeq Genomic Classifier (GC) applies a 74-gene DNA + RNA NGS panel to EUS-FNA cyst fluid and interrogates point mutations, copy-number changes, gene fusions, targeted expression shifts, and quantitative CEACAM5 mRNA in a single assay. In a multicenter training cohort of 108 cysts and an independent validation set of 77, a composite “cystic-precursor” score ≥ 3 identified IPMNs, MCNs, and IOPNs with 95% sensitivity and 100% specificity, while a “risk-of-advanced-neoplasia” score ≥ 4 detected high-grade dysplasia or incipient carcinoma with 82% sensitivity and 100% specificity [53]. These accuracies surpassed conventional markers such as fluid CEA, cytology, and imaging worrisome features; moreover, incorporating PancreaSeq GC as an additional criterion increased the sensitivity of both the IAP/Fukuoka and AGA guidelines by more than ten percentage points without eroding specificity. The assay also recognized cystic pancreatic NETs through CHGA overexpression and reliably excluded non-neoplastic cysts when no genomic alterations were found.

Within this framework, recent studies have investigated the role that certain genes may play in diagnostic terms, and so far, KRAS and GNAS have been the most studied among them [54,55,56,57,58,59]. The KRAS oncogene, located on chromosome 12p, is one of the most frequently mutated genes in pancreatic cancer, and its mutations are activating, leading to abnormal production of the protein of the gene. Over 90 percent of pancreatic cancers harbor a somatic KRAS mutation; furthermore, these mutations appear to occur very early in pancreatic carcinogenesis, as indicated by their presence in noninvasive precursors [60].

GNAS (GNAS Complex Locus) is located on chromosome 20q, and its continuous activation leads to constitutive receptor signaling and inappropriate production of excess cAMP.

The role of KRAS in the diagnosis of mucinous cysts was investigated in a meta-analysis conducted by Pfluger et al., where pooled analysis of six studies showed an overall high specificity of 99% but a moderate sensitivity of 61%. In the same meta-analysis, GNAS mutations were analyzed in four studies with 1071 cystic lesions, yielding a sensitivity of 44% and specificity of 100%. The two DNA-based biomarkers, when combined, improve sensitivity to 71% [61]. In the studies under review, GNAS also appeared to be associated with cystic lesions with a higher degree of dysplasia.

Recent molecular studies confirm that activating *GNAS* hotspot mutations (R201C/H) are virtually restricted to intraductal papillary mucinous neoplasms. Deep-sequencing of resected specimens shows a prevalence of ~70% overall—higher in the intestinal subtype—while the mutation is absent from most mucinous cystic neoplasms and other pancreatic cystic lesions [62,63]. Analysis of circulating cell-free DNA underscores this specificity: *GNAS* alleles were detected in 32% of IPMN patients versus 0% of non-IPMN cysts, with excellent concordance between plasma and tumor genotypes and a marked enrichment in intestinal-type IPMN [62]. Taken together, the presence of a *GNAS* mutation—whether in cyst fluid, tissue, or cfDNA—provides a highly specific molecular signature for IPMN and a practical discriminator from MCN and other non-mucinous entities. In this context, Schmitz et al. demonstrated that analyzing KRAS/GNAS mutations in EUS-FNA is a superior diagnostic tool compared to CEA and cytology for distinguishing mucinous from non-mucinous lesions, as shown in a study of 47 patients where mutation results were correlated with histopathology and clinical follow-up [64].

For serous cystadenomas (SCAs), Von Hippel–Lindau (VHL) mutations seem more likely to be detected; VHL mutations associated with SCAs were assessed in four studies (1140 cysts). These mutations achieved a pooled sensitivity of 56% and specificity of 99% for identifying SCAs, benign cysts with no malignant potential [61].

In addition, SCAs with VHL mutations and either TP53 or TERT mutations are associated with progressive pancreatic duct stricturing, leading to acute and chronic pancreatitis. While most SCAs are benign, asymptomatic, and slow-growing, a subset can exhibit increased growth and symptoms [65].

Increasing genomic evidence shows that activation of the MAP-kinase pathway in mucinous pancreatic cystic lesions can occur through BRAF rather than the canonical KRAS. While BRAF alterations are found in only ~2% of unselected PDAC [66,67], their frequency rises appreciably in cystic precursors that are wild-type for KRAS.

Resection-based deep-sequencing studies first documented BRAF mutations in 6% of high-grade IPMNs [60]. This signal was clarified in a prospective analysis of 108 EUS-FNA cyst fluids: among lesions molecularly classified as mucinous, 60% of the GNAS^+^/KRAS^−^ subgroup carried non-canonical in-frame insertions or deletions in exons 11 or 15 of BRAF (e.g., p.V600_K601delinsE, p.N486_P490del), with variant-allele fractions between 15% and 47%. Incorporating these BRAF hotspots into the NGS panel lifted the overall molecular sensitivity for mucinous cysts from 86.8% to 96% while maintaining 100% specificity [68]. Histological follow-up showed high-grade dysplasia or invasive carcinoma in three of five resected BRAF-mutant cysts, supporting a role in late-stage progression.

Clinically, cysts with the composite genotype GNAS^+^/KRAS^−^/BRAF^+^ often present as multifocal lesions with radiological, worrisome features and may merit intensified surveillance or earlier surgical resection. Therapeutically, analogous non-V600E BRAF alterations drive MAP-kinase signaling in the 2% of PDAC that are KRAS-wild-type; case series document partial responses to combined BRAF/MEK inhibition in this context [67,69]. Routine interrogation of BRAF exon 11/15 hotspots is therefore advisable whenever KRAS is wild-type, both to prevent false-negative molecular calls and to identify patients who could benefit from a targeted MAP-kinase blockade.

As we know now, mutations in other genes, such as CDKN2A, PIK3CA, SMAD4, and TP53, provide high specificity but are too insensitive to be useful alone for the diagnosis of mucinous cysts, as none of them have demonstrated a sensitivity higher than 20% [61].

Other types of molecular alterations have been assessed, like losses of heterozygosity of chromosome regions containing tumor suppressor genes known to be involved in specific cyst types or aneuploidy, which is known to increase with the grade of cyst dysplasia and with an associated invasive carcinoma with inconclusive results [56].

On the other hand, circulating tumor DNA (ctDNA), which is a tool used for monitoring tumor burden and early pancreatic cancer detection [70], has not yet found a place in the early identification and characterization of pancreatic cysts.

**Figure 2:**
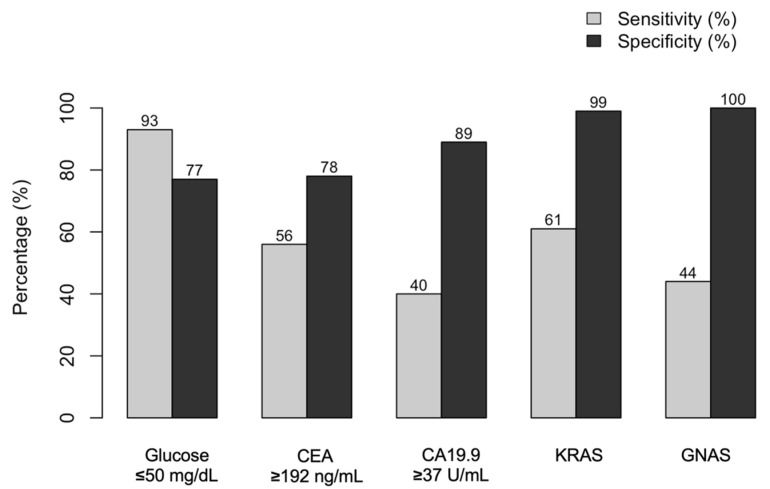
Sensitivity and specificity of different intracystic biomarkers for distinguishing mucinous from non-mucinous PCLs. The reported values derive from pooled data across multiple studies and meta-analyses.

Although these biomarkers are highly promising, the extremely low concentration of mutations in cyst fluid obtained via endoscopic aspiration poses a significant challenge for early detection, as illustrated in Figure 2 [50,51,61,64]. Advanced NGS techniques are required to reliably distinguish true somatic mutations from background noise, limiting their routine applicability in clinical practice [71].

Another significant limitation affecting NGS sensitivity for mucinous lesions stems from intrinsic features like epithelial atrophy and reduced cell turnover. These conditions, leading to decreased epithelial thickness and fewer cells shed into the cystic fluid, can substantially hinder lesion detection by NGS [62,63,72,73]. In this context, RNA- and proteomic-based analyses of cyst fluid could play a significant role in the detection and diagnosis of mucinous cysts. RNA-based analyses offer valuable insights by identifying gene expression profiles characteristic of mucinous cysts. For example, the integration of CEA mRNA (CEACAM5) into a combined DNA/RNA NGS platform has been shown to enhance the classification of pancreatic cysts and the detection of high-grade dysplasia and early adenocarcinoma [53]. Proteomic-based analyses, which involve the identification and quantification of proteins in cyst fluid, further contribute to diagnostic information. Proteomic profiling can identify specific proteins overexpressed in mucinous cysts, providing an additional layer of diagnostic utility. A study by Haeberle et al. demonstrated that molecular analysis of cyst fluids, including proteomic markers, improves the diagnostic accuracy of pre-operative assessments of pancreatic cystic lesions. This evidence suggests that combining RNA and proteomic analyses with NGS can significantly enhance the overall sensitivity and specificity for detecting and accurately diagnosing mucinous cysts [63].

## 6. Challenges and Future Directions

PCLs, especially those with premalignant or malignant potential, remain a diagnostic challenge. Although serum and intracystic biomarkers such as CEA and CA 19-9 have traditionally been used, their diagnostic accuracy in differentiating M from NM lesions is often suboptimal. By contrast, glucose measurement—especially the on-site evaluation—has emerged as a more accurate and cost-effective tool for this purpose. In parallel, DNA-based biomarkers such as KRAS and GNAS, though promising for their specificity, often lack the necessary sensitivity and are hindered by high costs, limiting their routine clinical use. These biomarkers may prove useful in managing cysts with worrisome features, either by prompting more aggressive treatment or by supporting closer monitoring, though further investigation is needed in these contexts. Despite their limited sensitivity, their specificity could help avoid unnecessary invasive procedures in patients with low clinical and radiological suspicion, particularly when surgery is risky due to comorbidities.

In procedural considerations, complete cyst evacuation during EUS-FNA remains controversial, as it enables more extensive biochemical and molecular analyses but disrupts the cyst’s native environment, potentially impairing future imaging and delaying detection of malignant transformation. The absence of standardized post-aspiration follow-up further raises concerns about both overuse of imaging and inadequate surveillance. In a setting where cytological analysis faces limitations in diagnostic yield and sample dependency, biomarker-centric approaches are driving more interest, prioritizing molecular and biochemical profiles.

EUS-guided needle-based confocal laser endomicroscopy (nCLE) offers a real-time “optical biopsy” by advancing a fluorescein-primed miniprobe through a 19-G needle into the cyst cavity. Characteristic image signatures—papillary fronds (IPMN), horizontal epithelial bands (MCN), and a superficial vascular network (SCN)—have been validated with sensitivities and specificities approaching 95–100% for distinguishing mucinous from non-mucinous lesions [74,75]. Independent cohorts confirm a diagnostic yield of ~84%, markedly superior to the traditional composite of cross-sectional imaging, cytology, and CEA [76,77,78]. Limitations remain—small prospective datasets, reader-dependent interpretation, and high procedural costs—so nCLE is not yet embedded in guideline algorithms; larger, cost-effectiveness studies are required before routine adoption in pancreatic cyst management.

While such strategies, including EUS-guided confocal laser endomicroscopy and molecular markers, hold promise for revolutionizing PCL management, further studies must validate the optimal thresholds, clarify the extent of cyst aspiration, and accurately identify populations most likely to benefit from genetic testing. A potential diagnostic algorithm could prioritize the on-site glucose evaluation as a first-line test for suspicious cysts undergoing EUS-FNA, with subsequent molecular testing (e.g., KRAS/GNAS) reserved for indeterminate or high-risk lesions. Integrating glucose testing with molecular tools may further refine diagnostic precision, minimize unnecessary resections, and detect malignant changes at earlier, more treatable stages.

It should also be noted that a “non-mucinous” classification does not necessarily preclude malignant potential or the presence of neoplastic changes. Certain lesions, such as cystic neuroendocrine tumors and solid pseudopapillary neoplasms, are categorized as non-mucinous yet may carry a significant risk of progression. While glucose determination appears potentially valuable in this setting, evidence remains limited, and further studies are needed to clarify its diagnostic impact for these less common yet clinically important lesions.

## Abbreviations

The following abbreviations are used in this manuscript:

CA 19-9	Carbohydrate Antigen 19-9
CEA	Carcinoembryonic Antigen
CT	Computed Tomography
EUS	Endoscopic Ultrasound
EUS-FNA	Endoscopic Ultrasound-Guided Fine Needle Aspiration
HGD	High-Grade Dysplasia
IPMN	Intraductal Papillary Mucinous Neoplasm
LD	Linear Dichroism
MCN	Mucinous Cystic Neoplasm
MDPI	Multidisciplinary Digital Publishing Institute
M-PCL	Mucinous Pancreatic Cystic Lesion
MRI	Magnetic Resonance Imaging
nCLE	Needle-based confocal laser endomicroscopy
NM-PCL	Non-Mucinous Pancreatic Cystic Lesion
NGS	Next-Generation Sequencing
PDAC	Pancreatic Ductal Adenocarcinoma
PCL	Pancreatic Cystic Lesion
SCN	Serous Cystadenoma
TLA	Three Letter Acronym
